# Policies and actions for electric vehicle battery waste processing using an integrated QFD approach: A case study for Jordan

**DOI:** 10.1016/j.heliyon.2025.e41940

**Published:** 2025-01-15

**Authors:** Fadwa Dababneh, Hussam Zuhair Aldababneh, Mohamad K. Khawaja, Rana Imam

**Affiliations:** a Industrial Engineering Department, German Jordanian University, Amman, 11180, Jordan; b Energy Engineering Department, German Jordanian University, Amman, 11180, Jordan; c Civil Engineering Department, The University of Jordan, Amman, 11942, Jordan

**Keywords:** Spent batteries, End-of-Life management, Waste management, Quality function deployment

## Abstract

Electric vehicles (EVs) are regarded as a key technology to fight climate change. However, with the increasing popularity of EVs, comes the challenge of handling spent EV batteries. Despite considerable research on end-of-life (EOL) methods, economics, and environmental benefits, many countries are facing challenges with the formulation of policies and proactive response actions needed for robust EOL infrastructure. There is a lack of research that employs comprehensive and systematic methods for identifying and prioritizing stakeholder needs, tradeoffs, and preferences. Accordingly, in this paper, a modified Quality Function Deployment approach is employed to prioritize actions, recommendations, and policies that address the handling of the spent batteries. The proposed method is applied on Jordan and a course of action and policies are proposed that will allow for the integration of adequate and appropriate EOL strategies. For Jordan, the most stringent needs revolve around the development of a collection center for EOL batteries and an incentive-based return process. Moreover, policy implications for the immediate, medium, and long-term horizons for Jordan were outlined.

## 1 Introduction

Electric Vehicles (EVs) are on the rise globally; in 2022, there were over 26 million EVs on the road, 60 % more than in 2021 [1]. Moreover, by the end of 2023, EV sales exceeded 14 million, representing a 35 % yearly increase [1]. With this increasing penetration of EVs, the environmental benefits from EVs, such as the reduction in greenhouse gas emissions, energy efficiency, reduced noise pollution, reduced reliance on fossil fuels, and the opportunity for more penetration of renewable energy, are heightened [2]. Research shows that a 5–10 % penetration of EVs results in a potential 45 % reduction in energy consumption and a 30 % reduction in greenhouse gas emissions [3]. Moreover, from a Life-Cycle Assessment (LCA) perspective, it was determined that life-cycle CO_2_ emissions associated with EVs are significantly lower than those of internal combustion engine vehicles (ICEV) [4].

Nonetheless, the increased adoption of EVs raises some challenges, specifically regarding EV battery waste at the national level. The waste accumulated from batteries that have reached their EOL introduces concern regarding the environmental risks associated with the handling of spent batteries. In particular, lithium-ion batteries (LIBs) are the most prevalent type of batteries embraced by EV manufacturers and are among the fastest spreading [5]. However, since lithium is very sensitive, LIBs are considered highly reactive when penetrated, exposed to water, subjected to high temperatures, etc. [6]. Nonetheless, at their EOL, LIBs are entering the waste stream and being diverted to landfills [7]. LIBs, when not discharged, can pose fire risks and have been reported to cause landfill fires [8]. Meanwhile, when subjected to landfill conditions such as crushing, exposure to sunlight, and rising pile temperatures caused by garbage decomposition risks are heightened [9].

Manufacturer warranties suggest that EV batteries will reach their EOL after approximately 8–10 years of use [10]. Disposing of spent batteries, typically by sending them to landfills, results in material loss, missed economic opportunity, and the aforementioned environmental and health hazards [11]. Several waste management strategies tackle these risks while also recovering value from spent batteries [12]. These strategies include remanufacturing, recycling, and repurposing [13]. Remanufacturing spent batteries allows for spent EV batteries that have a remaining battery capacity of less than 80 %, to be refurbished and then reused in automotive applications through repair and recovery methods. By assessing and inspecting the battery, faulty cells are identified and replaced with new cells, resulting in a functioning battery that can be reclaimed for automotive applications [14]. From a macro-level perspective, in one study, a closed-loop supply chain model was proposed and used to study the profitability of remanufacturing EV batteries; a 30 % increase in profit was observed when integrating remanufacturing into EV battery manufacturing supply chains [15]. Meanwhile, recycling provides the opportunity to recover valuable minerals from spent batteries. The four primary materials used in EV LIBs are cobalt, nickel, lithium, and aluminum oxides [16]. These materials are considered critical raw materials with significant economic value, and their supply is susceptible to supply chain interruptions [16]. Shafique et al. (2023) demonstrated that recycling spent LIBs has substantial economic potential and contributes to alleviating the demand-supply gap of raw materials for manufacturing EVs in Asia [17]. Lastly, EV batteries that have reached their EOL for vehicle applications may serve as a cost-effective alternative for energy applications in renewable energy storage systems [18]. Haram et al. (2021) reviewed the literature on repurposing spent LIBs and concluded that repurposing EV batteries in second-life applications has significant economic and environmental benefits [19].

In terms of maximizing the value of LIBs, remanufacturing or repurposing are more ideal compared to recycling [20]. Repurposing spent EV batteries generally has a lower carbon footprint compared to using lead-acid batteries or new EV batteries in energy storage [21]. Meanwhile, remanufacturing is commonly regarded as the most eco-friendly EOL strategy for a product [22,23]. Remanufacturing has also been found to enhance the sustainability of the energy sector, aiding the EV industry by reducing battery waste, and contributing to the development of charging infrastructure [24]. Moreover, reduction in greenhouse emissions is observed when recycling compared to production from virgin materials [25]. Leveraging these EOL strategies in a sustainable manner for EV LIBs could help promote sustainable production and consumption in the EV industry in alignment with the United Nation's (UN) sustainable development goal (SDG) 12. Moreover, the environmental benefits of EV batteries are underscored by their potential to be integrated with renewable energy for charging, reducing the use of fossil fuels, which is in line with the UN SDG goal 7 [26].

Each of the three waste management strategies for EV batteries has different characteristics and fulfills specific demand streams by promoting circular practices to achieve value recovery at various stages in the value chain. Even though there is plenty of research on EOL methods, principles, and practices, the main problem with implementing these EOL strategies comes from the lack of well-defined planning and regulatory policies. The successful implementation of such strategies must be done from a macrolevel and systematic perspective. This will require support at a national level through policy and infrastructure planning. Studies have been conducted on the relation of policies with circularity and waste management [[27], [28], [29]]. The need for mutually consistent policy interventions to achieve transparent management systems and effective stakeholder engagement in the waste sector is emphasized [27]. More specifically, studies on waste management policies for EV batteries have been conducted [30,31]. Albertsen et al. (2021) conducted an analysis of practices and policies for vehicle manufacturers in the EU and the adoption of circular economy strategies; their paper indicates that waste management strategies for EVs is context specific and depend on internal factors [30]. Another paper by Malinauskaite et al. (2021) also highlights that further policy development is necessary to ensure compliance with the waste hierarchy. Meanwhile, the need for interdisciplinary solutions to manage the EOL of EV batteries to prevent future waste issues is addressed and current EU and UK frameworks, and policies for battery waste management are critiqued [31]. Recent developments in the EU, including a proposal for a new EU Batteries Regulation and the impact of Brexit on the UK's future policy direction in this area are examined [31].

To better understand the state of battery waste management, it is important to outline the status quo across various regions. Nurdiawati et al. (2022), outlined the process for managing EOL LIBs in Sweden; where batteries are collected and pre-processed by El-Kretsen, a nationally approved system that handles waste electronics and batteries [32]. Kishita et al. (2024) describe multiple approaches to managing EOL management of batteries in Germany, Norway, and Japan, where the Original Equipment Manufacturers (OEMs) in Germany are responsible for covering the costs of collecting and recycling batteries. Meanwhile, in Norway, retailers are responsible for collecting used EV batteries and delivering them to authorized waste facilities. On the other hand, in Japan despite the automobile recycling law that mandates the recovery of batteries from dismantled EVs for material recycling, there lacks national policies specific to the collection of EV batteries [33]. Additionally, Mathew et al. (2023) described that the market in Malaysia for LIBs is driven by consumer electronics such as smartphones, laptops, and smartwatches these devices dominate LIBs applications and that the demand for LIBs is rising [34]. Moossa et al. (2023) highlighted the absence of formal e-waste management frameworks across the MENA region. Stating that this is mainly due to a lack of policies, infrastructure, and institutional frameworks, resulting in the informal handling and export of batteries, as seen in countries such as Bahrain, Egypt, and Iran, where e-waste is exported or disposed of improperly [35]. Meanwhile, Alhakbani (2020) argued that the urgency for establishing domestic recycling infrastructure in Saudi Arabia does not align with immediate strategic interests due to the current lack of local cell manufacturing and the relatively low adoption of EVs. Hence, developing recycling infrastructure is considered less attractive at this point for Saudi Arabia [36]. On the contrary, Almarzooqi et al. (2019) highlighted the fast adoption of EVs in Dubai which could generate a significant number of LIBs waste that need to be recycled, hence this adoption presents various challenges in waste management, particularly related to developing efficient recycling infrastructure in the near future [37]. Finally, Khawaja et al. (2019) described the status of energy storage system waste in Jordan. The authors highlight that energy storage system waste is regulated by the Ministry of the Environment (MoE) under Environmental Protection Law No. 52 of 2006, which highlights that batteries are meant to be disposed of at the Swaqa hazardous waste landfill. Nonetheless, most primary batteries end up in regular landfills or are incinerated due to insufficient collection bins and a lack of enforcement for proper battery waste separation [38].

While studies show that policy and action plan development are essential for successful EOL management, methods for formulating and prioritizing policies and actions to facilitate the proper handling of spent EV batteries while considering stakeholder needs are lacking. Accordingly, in this paper, a Quality Function Deployment (QFD) method, that integrates Analytical Hierarchy Process (AHP) prioritization, is proposed to translate stakeholder needs into action plans and policy recommendations. The QFD method is traditionally used to translate the voice of the customer (VOC) to technical requirements at different stages of the product development process, such that the goal is to ultimately increase the satisfaction of the customer/user for the product [39]. Accordingly, QFD is highly beneficial to understand and develop strategies that differentiate and give competitive advantages by maximizing quality and meeting customer expectations [40]. The standard QFD approach involves four phases. These phases are performed by utilizing the house of quality (HOQ). Although QFD has been mainly designed and used as a tool for product development, various modifications and approaches to QFD have been investigated and implemented [[41], [42], [43], [44], [45], [46]]. An et al. (2008) developed an integrated product-service roadmap using a modified QFD approach to aid in strategic planning and management of product-service systems [41]. Next, Vimal et al. (2021) introduced a new Remanufacturing Quality Function Deployment (RQFD) methodology, which integrated traditional QFD with sustainability and remanufacturing strategies [42]. Moreover, a multi-stakeholder policy QFD method was developed, integrating traditional QFD with AHP in order to align diverse stakeholders’ interests with policy developments [44]. Based on the literature on QFD, it can be seen that QFD methods help combine insights of various stakeholders (in place of “customers”) and tactical needs, to guide policy and strategy recommendations that promote circularity and sustainable EOL management of EV LIBs.

While there has been much research on waste management challenges and EOL strategies for spent EV batteries, there is a notable gap in the policy development methods that integrate stakeholders' needs to guide national roadmaps and recommendations for the handling of EV battery waste. Therefore, this paper proposes a method to prioritize actions and policies for the management of EOL EV batteries, using a modified QFD approach that translates stakeholders’ requirements into policies and infrastructure recommendations. The aim is to foster a sustainable ecosystem for handling spent EV batteries. Accordingly, the rest of the paper is organized as follows: in Section 2, the proposed methodology and the modeling approach is outlined; in Section 3, the results and discussion for a case study focused on Jordan will be presented; in Section 4, the policy implications are summarized; and, lastly, in Section 5 the conclusions are drawn, and future work directions are identified and discussed.

## 2 Methodology

The first stage in the methodology is to define the current waste management processes and procedures. This is followed by identifying stakeholders and translating their requirements into the “voice” of the stakeholder (VOS). Next, it is necessary to determine candidate waste management strategies that could be implemented. Afterward, data collection, for gathering qualitative and quantitative data, must be conducted and used to calculate scores for various comparative criteria. In turn, the scores are then used to guide the evaluation of the stakeholders’ requirements alongside potential waste management strategies.

**Fig. 1:**
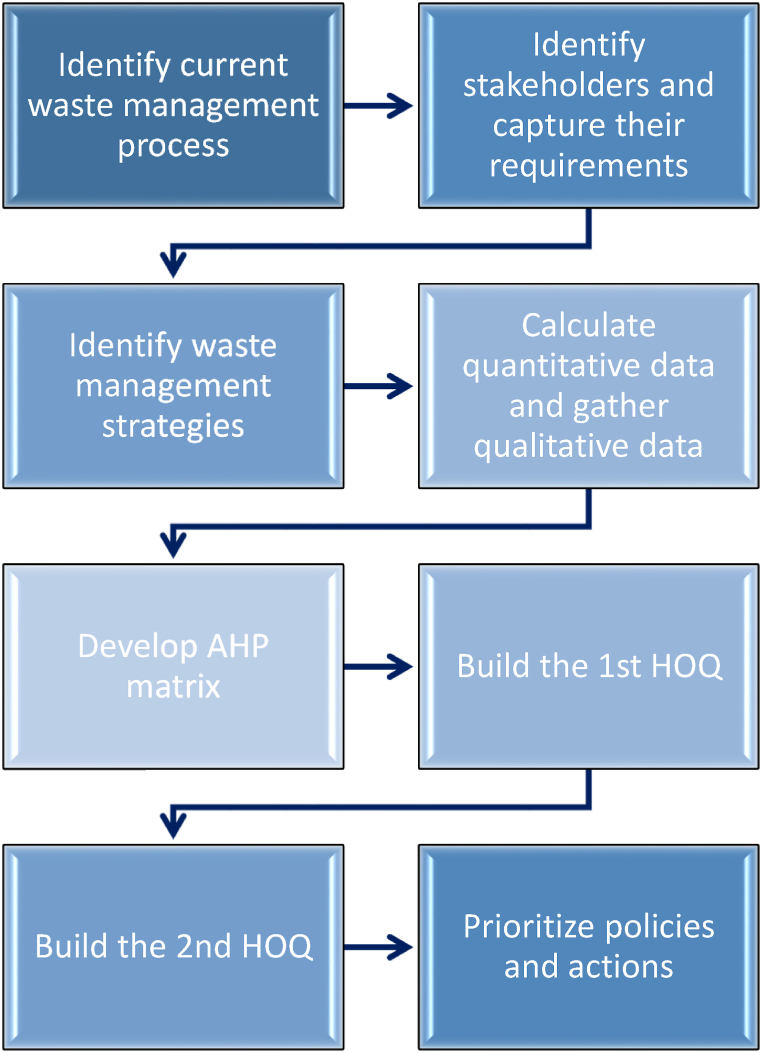
Methodological framework structure.

For the determination of the status quo waste management processes, stakeholder requirements, and EV battery data need to be obtained from various sources such as expert interviews, research publications, news articles, governmental reports, and historical records. Next, an AHP matrix must be developed, which evaluates and compares stakeholders’ requirements based on importance, effectively determining the relative vitality of each requirement. Following this, the first HOQ must be built, aligning the stakeholders' requirements with the waste management strategies and establishing the rank and weight of each strategy. Afterward, the second HOQ is needed to map waste management strategies to specific actions and policies, allowing for the prioritization of actions and policies through the obtained ranks and weights. Finally, policies and actions can be prioritized based on the insights obtained from the second HOQ; policies and actions are categorized based on their priority levels – high, medium, or low. The outline for the proposed method is illustrated in Fig. 1.

### 2.1 Modelling approach for criteria assessment

Several criteria can be used to assess different waste management strategies to guide the judgment for the QFD approach. Among these criteria is the revenue potential from implementing the different EOL strategies. For the revenue potential, first the forecast of spent EV batteries quantities is needed. To forecast the number of spent EV batteries entering the waste stream, the number of EVs entering the transportation fleet can be used. Accordingly, a moving average method with an adjustment factor to account for national EV penetration objectives can be used as shown in Equation (1),

(1) $${EV}_{i,t}=\frac{\sum_{t-w}^{t-1}{EV}_{i,t}}{w}\times \left(1+r\right)$$

where *EV*_*i,t*_ represents the number of EVs of type *i* in year *t*, *r* represents the percentage increase, and *w* represents the moving average window. Meanwhile, Equation (2) projects the number of spent EV batteries,

(2) $$Y_{spent,n,i}=\left(Y_{entry,n,i}+X_{n}\right)-L_{n}$$

where *Y*_*spent,n,i*_ is the year in which EV battery *n* of type *i* is considered to be spent; *Y*_*entry,n,i*_ is the vehicle's model year *n* of battery type *i* ; *X*_*n*_ is the age correction factor of EV battery *n* to account for lags between the model year and the year the vehicle enters the transportation fleet; and *L*_*n*_ is the estimated life of battery *n*.

Next, SB, in Equation (3), is a binary variable representing whether *Y*_*spent,n,i*_ is equal to year *t* or not. Moreover, the number of spent EV batteries is denoted by *EVB*_*i,t*_, which is the sum of the number of spent EV batteries of type *i* that belong to year *t*. This is shown in Equation (4).

(3) $${SB}_{n,i,t}=\left\{\begin{array}{cc} 1 & Y_{spent,n,i}=t\\ 0 & otherwise \end{array}\right\}$$

(4) $${EVB}_{i,t}=\sum_{n=1}^{N}{SB}_{n,i,t}$$

Subsequently, the revenue for remanufacturing EV batteries over the decided time period *t* is formulated in Equation (5), utilizing the forecast of spent batteries,

(5) $${REV}_{remanufacturing}=\sum_{t\in T}\sum_{i\in Types}{EVB}_{i,t}\times \text{RM}\times {SC}_{i}$$

where *REV*_*remanufacturing*_ is the total remanufacturing revenue, *RM* is a parameter representing the expected percentage of batteries that are suitable for remanufacturing, and *SC*_*i*_ is the selling cost of remanufactured battery of type *i*.

The revenue for recycling is formulated in Equation (6)

(6) $${REV}_{recycling}=E_{f}\times \sum_{t\in T}\sum_{i\in Types}\left({EVB}_{i,t}\times W_{i}\times \sum_{m\in minerals}P_{m,i}\times {MC}_{m}\right)$$

where *REV*_*recycling*_ is the total recycling revenue over the decided time period *t*, *E*_*f*_ is a parameter for the expected efficiency of the recycling process, *W*_*i*_ is the weight of battery type *i*, *P*_*m,i*_ is a parameter representing the percentage of material (i.e. minerals) the battery is composed of, where *m* is index for the recyclable material, and *MC*_*m*_ is the cost of mineral *m*.

Lastly, the repurposing revenue, *REV*_*repurposing*_*,* is formulated in Equation (7), where *R*_*f*_ is the percentage of batteries to be repurposed, *SP* is the price per unit power of repurposed EV batteries, and *BC*_*i,n*_ is the baseline battery capacity for battery type *i*.

(7) $${REV}_{repurposing}=R_{f}\times \left[\sum_{n\in N}\sum_{t\in T}\sum_{i\in Types}{EVB}_{i,t}\times SP\times {BC}_{i,n}\right]$$

### 2.2 Analytical Hierarchy Process overview

The revenue potential modeled in the previous section will be supplemented with different criteria based on national needs and stakeholder objectives; to determine the necessary priorities and action plans needed to support sustainable EOL management of EV batteries at a national level. Accordingly, an AHP dominance matrix can be used to determine the priority and ranking of stakeholders' requirements and needs. AHP is a mathematical technique utilized for assigning weights to criteria through pairwise comparisons and for assessing the relative significance of each criterion [47]. The stakeholders’ requirements are placed in a pairwise comparison matrix, *p*_*kj*_, and aggregated based on criteria importance rating.

(8) $$p_{kj}=\left[\begin{array}{cccc} 1 & a_{12} & \ldots & a_{1j}\\ \frac{1}{a_{12}} & \ddots & \ldots & a_{2j}\\ \vdots & \vdots & \ddots & \vdots \\ \frac{1}{a_{1j}} & \frac{1}{a_{2j}} & \ldots & 1 \end{array}\right]$$

The requirements are then scored, where *a*_*kj*_ represents the importance of requirement *k* over requirement *j* as displayed in Equation (8). Each column of the matrix is normalized as formulated in Equation (9)

(9) $${Nv}_{kj}=\frac{Me_{kj}}{\sum_{i=1}^{\eta }Me_{kj}}$$

where *Nv*_*kj*_ is the normalized value of the element in the *k*th row and *j*th column of the matrix, *Me*_*kj*_ is the original value in the *k*th row and *j*th column of the comparison matrix, *η* is the number of requirements being compared, and $\sum_{k=1}^{\eta }Me_{kj}$ is the sum of all elements in the *j*th column of the matrix.

Following the normalization, the weight for each stakeholder requirement is calculated in Equation (10) by averaging the values of each row of the normalized matrix, where *W*_*k*_ is the weight for each requirement.

(10) $$W_{ik}=\frac{\sum_{j=1}^{\eta }{Nv}_{kj}}{\eta }$$

### 2.3 Quality Function Deployment

**Fig. 2:**
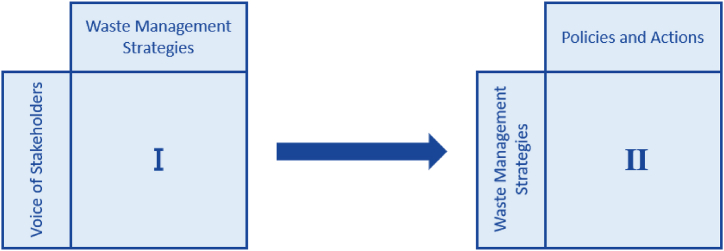
Phases of the proposed QFD approach.

Meanwhile, the QFD approach is also used and considers two phases. In the first HOQ, the requirements of the stakeholders are assessed against waste management strategies, while in the second HOQ strategies are translated into policies and actions, as shown in Fig. 2.

The weight for each stakeholder requirement will be utilized as the importance ranking for each requirement in the first HOQ. The stakeholder needs are placed in rows in the first HOQ and the waste management strategies as columns. Based on the quantitative and qualitative data collected, each strategy was assessed based on the stakeholder need, utilizing a scale of 0,1,3 and 9. 0 means no relationship between the stakeholder requirement and the waste management strategy, 1 if the relationship is weak, 3 if medium, and 9 if the relationship is strong.

The absolute weight for each strategy can be calculated using Equation (11)

(11) $${Absw}_{j}=\sum_{k=1}^{\eta }\mu_{k}\times {Rt}_{kj}$$

where *Absw*_*j*_ is the absolute weight of *j*th column, *μ*_*k*_ is the weight of *k*th row, and *Rt*_*kj*_ is the relationship rating between *k*th row and *j*th column.

The relative weight formulation is expressed in Equation (12), where the relative weight for each strategy is calculated by dividing the absolute weight over the sum weight of all of the columns,

(12) $${Relw}_{j}=\frac{{Absw}_{j}}{\sum_{j=1}^{\eta }{Absw}_{j}}$$

*Relw*_*j*_ is the relative weight of the *k*th column and *η* is the total number of columns.

Afterward, the second HOQ can be built, and the relative weight for the waste management strategies from the first HOQ can be used for the ranking in the second HOQ. The policies and actions for waste management of EV batteries are then portrayed in the column section of the HOQ, whereas the row section consists of the EV battery's waste management strategies.

## 3 Results and discussion

The proposed method was applied to the country of Jordan. The current waste management procedure for EOL batteries was identified by conducting expert interviews with governmental agencies and responsible entities [48]. Accordingly, a clear image of the current handling process of spent EV batteries was established. Through the interviews, the essential stakeholders were identified, and the needs of the stakeholders were captured. The waste management strategies were considered and an AHP matrix was developed to provide the ranking for each stakeholder's needs. Utilizing the modified QFD approach, policies, and actions were prioritized.

Expert interviews consisted of in-person and online interviews with stakeholder representatives from the Jordanian Ministry of the Environment, the Jordan Import Customs Department, the Jordan Vehicle License and Registration Department, NGOs, EV mechanics, private sector entities, etc. Meanwhile, news articles and governmental reports outlined import requirements and tariff percentages, environmental policy development, and national strategic goals. For example, the Official Gazette Issue No. 5929 outlined import requirements for EVs related regulations and safety compliance [49]. Meanwhile, the tariff percentages are published by the Prime Ministry [50] and environmental policy development and disposal regulations are published by the Ministry of Environment [51]. Finally, national strategic goals were referred to from the Green Growth National Action Plan [52]. As for historical records, EV battery quantities, types, and characteristics were obtained from the Jordan Import Customs Department and the Jordan Vehicle License and Registration Department [53].

### 3.1 Status quo in Jordan

**Fig. 3:**
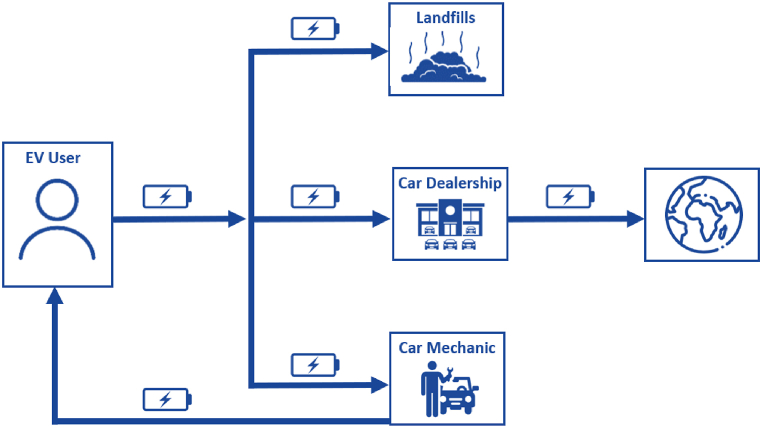
Current options for handling spent EV batteries in Jordan.

EV batteries that have reached their EOL in Jordan are either handled by mechanics, to be refurbished for reuse by the same EV owner, or by car dealerships, where they are exported outside of the country or sent to a landfill in which they are stored. The available options for consumers to handle spent EV batteries are listed in Fig. 3. When batteries are repaired by mechanics, another replacement, within a short time period, may be required if the repair was done inadequately. Moreover, the return of spent batteries to dealerships is not certain and dependent on the role and purchase agreement signed with the dealership. Hence, the Swaqa landfill (Jordan's hazardous waste landfill) is the primary location where these spent batteries are disposed of. Unfortunately, there are no designated sites for discarding EV batteries within the Swaqa landfill. The Jordanian Ministry of Environment requires EV owners to submit a personal pledge to return the battery after its retirement so that it can be disposed of safely in the Swaqa landfill [54]. Nonetheless, there is a clear lack of policies and procedures for the proper disposal of EV batteries in landfills, which renders the return and tracking of EV batteries challenging and inconsistent. Nonetheless, according to the Jordan Customs Annual Reports, a compelling focus for the increasing adoption and associated economic benefits of EVs is highlighted. Accordingly, the economic value of EVs surged from 77.5 million Jordanian Dinars (JOD) in 2019 to 696.2 million JOD in 2023, reflecting a remarkable growth of approximately 798 % [53], which is expected to have a similar impact on the market value of spent EV batteries.

The identified stakeholders involved in the procedure of handling EV battery waste are categorized into current and potential stakeholders. Current stakeholders include governmental agencies, EV owners, car dealerships, and repair shops. Potential stakeholders include collection centers, repurposers, remanufacturers, and recyclers. The criteria and requirements that are of the most concern for the stakeholders were identified and collected include: satisfying customers’ needs for EV battery replacements, minimizing waste, revenue potential of the waste management approach, positive environmental impact, and the promotion and advancement of green jobs opportunities.

Remanufacturing, recycling, and repurposing will be considered and are summarized accordingly. In remanufacturing, spent batteries are sent to collection centers, which are then transferred to a remanufacturing facility for refurbishing. Remanufacturing the spent batteries results in batteries that are suitable for automotive applications again. This allows EV owners to obtain batteries sourced in Jordan when their vehicles require battery replacements. For recycling, spent EV batteries are also collected by collection centers and sent to a recycling facility where minerals such as lithium, nickel, and cobalt are recovered. The recovered minerals are either used locally or exported outside the country. Repurposing entails the distribution of spent EV batteries to collection centers and then the transfer to a repurposing facility. In the repurposing facility, the batteries are adapted for different uses in second-life applications such as for grid energy storage. Remanufacturing meets the EV users’ needs by producing EV batteries that can be used to replace EV batteries that have reached their EOL, whereas repurposing and recycling do not. Not all spent EV batteries have a sufficient state of health (SoH) to be remanufactured and repurposed, but in recycling more spent batteries are processed providing a higher waste reduction potential.

### 3.2 Data and parameter values

**Fig. 4:**
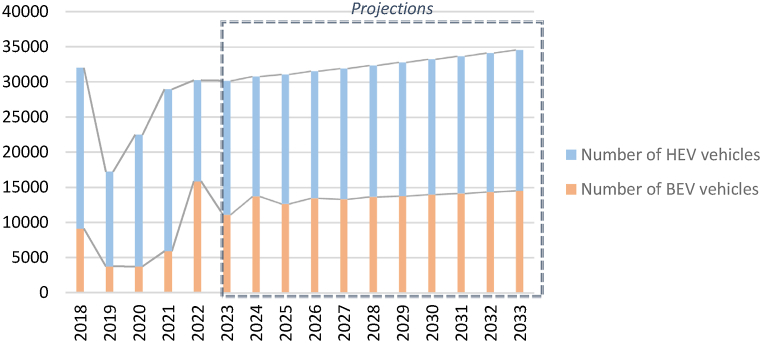
Projection of EVs in Jordan until 2033.

The data was obtained from various entities such as the Customs Department in Jordan. The following data limitations should be noted. Only data on EV imports from 2018 to 2022 was available for the study. Moreover, the data obtained did not provide information on the model year or model category of each vehicle; as well as, the EV battery's age, nameplate capacity, and number of modules/cells. As for the battery composition, since data at the battery composition level was not available, a standard battery composition for nickel, lithium-ion, etc. was assumed. Lastly, for this study, it is assumed that the batteries are all LIBS batteries since the technological trend for EV batteries is moving in this direction. Considering this, the projections for the number of EVs in Jordan until the year 2033 is illustrated in Fig. 4. The number of hybrid electric vehicles (HEVs) and battery electric vehicles (BEVs) is generally increasing from 2021 to 2033. The drop in 2019 and 2020 could be attributed to the COVID-19 pandemic.

**Fig. 5:**
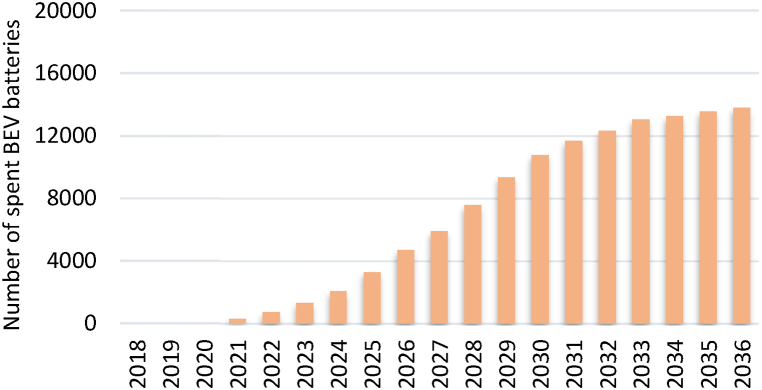
Projection of the number of spent BEVs batteries.

**Fig. 6:**
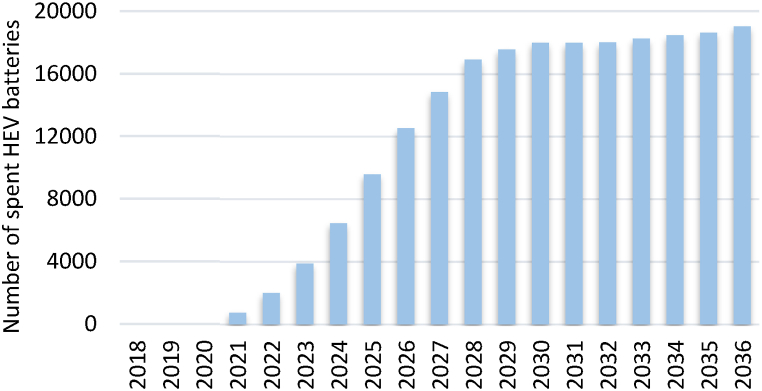
Projection of the number of spent HEVs batteries.

Fig. 5, Fig. 6 outline the forecast of the number of spent BEV and HEV batteries, respectively, from 2018 to 2036 based on the projection of EVs. This forecast estimates the amount of EV battery waste that Jordan will have to handle. Meanwhile, the values of the parameters used to calculate the revenues are listed in Table 1.

**Table 1:** Parameters and values for forecasting and projections.

Parameter	Value	Source
*RM*: percentage of batteries suitable for remanufacturing	85 %	
*SC*_*HEV*_: selling cost of remanufactured HEV battery	3125 USD	[55]
*SC*_*BEV*_: selling cost of remanufactured BEV battery	6250 USD	[56]
*W*_*HEV*_: weight of HEV battery in kg	125 kg	[57]
*W*_*BEV*_: weight of BEV battery in kg	475 kg	[57]
*P*_*nikel*_: percentage of nickel in battery	15.7 %	[58]
*P*_*cobalt*_: percentage of cobalt in battery	4.3 %	[58]
*P*_*lithium*_: percentage of lithium in battery	3.2 %	[58]
*MC*_*nikel*_: cost of nickel (USD/kg)	12.6 USD/kg	[59]
*MC*_*lithium*_: cost of lithium (USD/kg)	20 USD/kg	[60]
*MC*_*coblt*_: cost of cobalt (USD/kg)	27.5 USD/kg	[61]
*E*_*f*_: recycling efficiency	65 %	[62]
*SP*: selling price of repurposed battery (USD/kWh)	100 USD/kWh	[63]
*BC*_*HEV*_: HEV battery capacity (kWh)	8 kWh	[64]
*BC*_*BEV*_: BEV battery capacity (kWh)	40 kWh	[65]
*R*_*f*_: percentage of batteries to be repurposed	50 %	

**Fig. 7:**
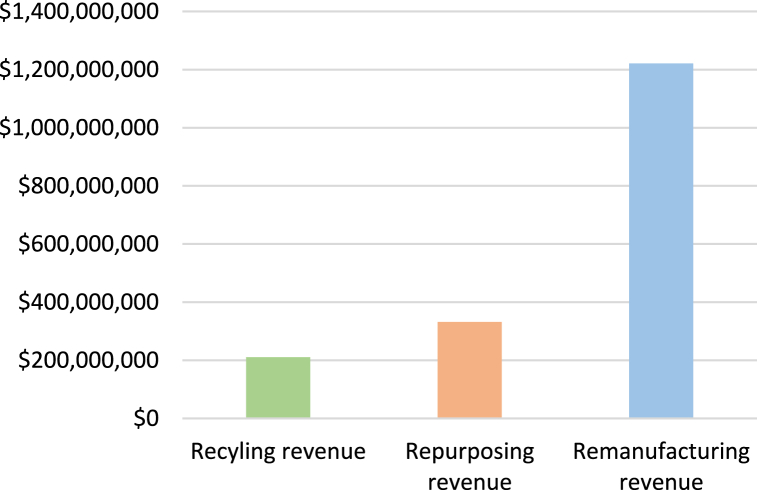
EOL EV battery strategies revenue in Jordan over a 16-year period.

An analysis period of 16 years (from 2021 to 2036) was selected based on the cyclic nature of the forecasting method used and due to limited data. Over the 16-year period, the projected revenue from remanufacturing EV batteries in Jordan is 1.2 billion USD, the projected recycling revenue is 210 million USD and the projected repurposing revenue is 332 million USD as shown in Fig. 7. This revenue would enter the Jordanian economy if repurposing, remanufacturing, or recycling facilities become available and operational in the country. The results show that recycling does not yield significant revenue potential. Since the extraction of materials (such as lithium) from batteries may not accumulate to significant quantities due to the relatively small number of spent batteries in Jordan compared to what is needed to justify recycling infrastructure, the revenue potential yielded by recycling is not considered substantial. Moreover, repurposing may be subject to challenges related to procurement for stationary energy storage applications. Lastly, remanufacturing shows to provide significant revenue potential and may be a promising investment opportunity in Jordan since procurement of these batteries will be distributed over a greater pool of consumers, however, requires much policy and infrastructure support.

### 3.3 Developing the Analytic Hierarchy Process (AHP) matrix

In-person and online interviews that gathered expert insights on major influencers of sustainable life cycles of EV batteries in Jordan. Meanwhile, weights were set based on a combination of subjective and quantitative support derived from in-person and online interviews. Next, the Analytic Hierarchy Process was used for the pairwise comparison with the normalization and the relative weighting listed in Table 2. By assessing the stakeholders’ requirements relative to each other, it is observed that revenue has the highest relative weight and, therefore, the highest priority, followed by consumer needs, waste reduction, positive environmental impact, and green jobs promotion.

**Table 2:** Normalized AHP matrix with relative weight.

Requirements	EV user needs	Revenue	Positive environmental impact	Waste reduction	Green jobs promotion	Relative Weight
**EV user needs**	0.21	0.18	0.24	0.31	0.24	0.24
**Revenue**	0.62	0.54	0.41	0.52	0.43	0.50
**Positive environmental impact**	0.07	0.11	0.08	0.03	0.14	0.09
**Waste reduction**	0.07	0.11	0.24	0.10	0.14	0.13
**Green jobs promotion**	0.04	0.06	0.03	0.03	0.05	0.04

### 3.4 Implementing QFD and prioritizing actions and policies

Next, the QFD method is employed to prioritize actions and policies needed to support Jordan. The first HOQ constructed is shown in Table 3, remanufacturing has the highest relative ranking of 0.63, repurposing has a ranking of 0.21, and recycling has the lowest relative ranking of 0.16. This means that remanufacturing is a strategy that aligns the most with the requirements of the stakeholders followed by repurposing and recycling.

**Table 3:** First house of quality (HOQ).

Requirements	Weights	Strategies		
		Remanufacture	Recycle	Repurpose
EV user needs	0.24	9	0	0
Revenue	0.50	9	1	3
Positive environmental impact	0.09	9	3	9
Waste reduction	0.13	3	9	3
Green jobs promotion	0.04	9	3	3
	Total Weight	8.20	2.09	2.82
	Ranking	0.63	0.16	0.21

Next, policies and actions to be implemented were identified as: establishing collection centers, opening a remanufacturing plant, opening a recycling plant, opening a repurposing facility, implementing traceability, establishing a database for EV batteries, training, certification, licensing for high voltage technicians working on EV batteries, and an incentive system for returning EV batteries by EV users. The actions and policies were given acronyms as summarized in Table 4.

**Table 4:** Acronyms for actions and policies.

Acronym	Action/Policy
ECC	Establishing a collection center
RMP	Opening a remanufacturing plant
RCP	Opening a recycling plant
RPF	Opening a repurposing facility
TTD	Implementing traceability of EV batteries and establishing a database for EV batteries
TCL	Training, certification, and licensing for high-voltage technicians
RIC	Return incentive system for EV batteries by EV users

Afterward, the second HOQ is constructed and summarized in Table 5. According to the rankings determined by the second HOQ for the policies and actions, categorization based on ranking into high, medium, or low priority are listed in Table 6.

**Table 5:** Second house of quality (HOQ).

Strategies	Weights	Actions and policies						
		ECC	RMP	RCP	RPF	TTD	TCL	RIC
Remanufacturing	0.63	9	9	0	0	3	9	3
Repurposing	0.21	9	0	0	9	3	3	3
Recycling	0.16	9	0	9	0	1	3	3
	Total Weight	8.91	5.58	1.44	1.89	2.65	6.69	2.97
	Ranking	0.30	0.19	0.05	0.06	0.09	0.22	0.1

**Table 6:** Prioritization of actions and policies.

Rank	Action/Policy	Priority
1	Establishing a collection center	High
2	Training, certification, and licensing for high-voltage technicians	High
3	Opening a remanufacturing plant	High
4	Return incentive system for EV batteries by EV users	Medium
5	Implementing traceability of EV batteries and establishing a database for EV batteries	Medium
6	Opening a repurposing facility	Low
7	Opening a recycling plant	Low

The highest priority was to establish a collection center for EOL batteries. The EV batteries arrive at collection centers when they have reached their EOL and are sorted based on their model, type, quality level, and safety assessment tests. Performing training and establishing certification and licensing for high voltage technicians is essential to minimize safety issues and to designate safe spaces for operating on spent EV batteries. Opening a remanufacturing plant was also deemed as a high priority where, after the collection center, spent EV batteries will be transferred to a remanufacturing plant based on their SoH. Spent EV batteries will be refurbished and used in the local market for EV battery replacements.

A return incentive system will encourage EV users to return their depleted EV batteries to collection centers by offering a monetary incentive or rebates for return as suggested in public-private partnerships [66]. Meanwhile, implementing tracking and traceability ensures the identification, documentation, and monitoring of EV batteries, it enables the safe and reliable handling of EV battery waste by tracing batteries and determining their previous path and usage and by tracking them to ensure they are undergoing the appropriate waste management procedure. All this information should be stored on a central database for easy and efficient access. Moreover, while repurposing and recycling facilities are beneficial to the waste management of EV batteries; the study shows that implementing remanufacturing is currently more urgent in Jordan. In all, effectively prioritizing actions and policies to manage EV waste is essential. This approach provides valuable insights to help establish an environment that tackles EV battery waste issues promoting long-term sustainability and safety while considering the requirements of key stakeholders in Jordan.

## 4 Policy implications

To effectively implement the findings the following policy implications are needed. In the immediate term, the Jordanian government needs to develop a national strategy for spent battery collection that includes robust return/collection schemes, storage infrastructure, and data tracking mandates. For this to work, roles and responsibilities along with a regulatory framework are needed for the safe and environmentally conscious collection, handling, storage, and transportation of spent batteries. The Ministry of Environment should lead the EOL management for spent batteries since spent batteries fall under the hazardous waste classification. Nonetheless, the Ministry of Environment must work with the Ministries of Industry and Energy, as well as with Import customs and local municipalities to align policies and identify gaps. Meanwhile, to support infrastructure development, subsidies for the establishment of collection centers are needed along with public-private sector partnerships. Incentive schemes tailored to EV battery owners to return their spent batteries are needed. Moreover, Extended Producer Responsibility (EPR) should be enforced to ensure proper disposal and collection of spent batteries by automotive distributors.

In the medium-term, the Jordanian government needs to develop policy frameworks and support mechanisms that help Jordan to shift to a circular economy model for spent batteries. Industries such as refurbishment, remanufacturing, and repurposing must be encouraged. Once again, incentives, such as tax breaks or grants, for the emergence of such industries are needed. Meanwhile, data reporting should be mandated and made available and accessible to provide investors and policymakers visibility into supply and demand streams. The Ministry of Industry should play a primal role in ensuring an enabling environment for such activities. Furthermore, the Jordan Standards and Metrology Department should ensure compliance with safety and operational standards for second-life batteries while considering SoH conditions needed for various second-life applications.

Lastly, in the long-term, policies should push for the introduction of recycling by providing a legal structure for stakeholders. Moreover, investment in infrastructure is needed and can be encouraged through financial incentives. Generally, policies should be adaptable and keep up with technological trends and changing technology for EV battery recycling, as this is an industry that is still being researched and developed. In all, clearly defined policies and regulatory frameworks from the Jordanian government can lead to a clearer understanding of activities needed to translate Jordan's EV battery waste into a valuable revenue stream.

## 5 Conclusion and future work

In this paper, an approach for the prioritization and guidance of policy and actions for EV batteries waste management strategies is proposed, the approach utilizes a modified QFD approach to design actions and policies for waste handling of EV batteries by identifying current waste handling processes, evaluating key stakeholders’ needs, and setting priorities. The methodology was applied to Jordan where there is a noticeable lack of infrastructure and poor handling of spent EV batteries. The high-priority actions and policies that were identified for Jordan include the establishment of collection centers for storing retrieved spent EV batteries, as opposed to landfills where they are currently disposed of. The approach also identifies training, licensure, and certification for EV battery operators as a high priority to ensure safe handling. Furthermore, developing a remanufacturing plant to refurbish spent EV batteries to a level that is suitable for reuse in EVs is necessary to satisfy the needs of the stakeholders. Accordingly, policy implications for the immediate, medium, and long-term horizons for Jordan are outlined. With suitable policies and actions for the circularity of spent batteries in Jordan, many social and economic advantages can be gained. These include consumer benefits through the promotion of locally processed and less expensive battery alternatives. Moreover, green job creation opportunities will arise. Finally, regional collaboration and trade opportunities between Jordan and the surrounding region will be stimulated, which will contribute to the economic development of the country.

The following future work directions are noted. First, it could be valuable to look at regional-level frameworks for EV battery waste management that take into account challenges and collaboration opportunities in the MENA region. When adopting a regional approach, methods that robustly process subjective and varying stakeholder and government needs and perspectives are needed. Fuzzy sets, which deal with reasoning that is approximate rather than fixed and exact, could enhance the robustness of judgments, particularly in areas with ambiguous or uncertain data. The integration of fuzzy logic into the evaluation process would allow for a more robust assessment of stakeholder needs. Next, mechanisms for tracking and tracing imported batteries whether in EVs or standalone batteries are needed. Hence, tracking and traceability technical requirements must be defined and a method for integrating them into already existing regulatory and business frameworks is needed. Moreover, different incentive schemes could be investigated and modeled to study the willingness of EV owners to properly dispose of their EOL EV batteries. Finally, it is important to perform a comprehensive cost-benefit analysis and a feasibility analysis for waste processing, refurbishment, remanufacturing, and repurposing considering the unique challenges of developing countries with limited access to limited resources.

## CRediT authorship contribution statement

**Fadwa Dababneh:** Writing – original draft, Supervision, Methodology, Conceptualization. **Hussam Zuhair Aldababneh:** Writing – original draft, Methodology. **Mohamad K. Khawaja:** Writing – review & editing, Conceptualization. **Rana Imam:** Writing – review & editing, Conceptualization.

## Data availability

Data included in the article material is referenced in the article.

## Declaration of competing interest

The authors declare that they have no known competing financial interests or personal relationships that could have appeared to influence the work reported in this paper.
